# Knowledge, attitude and practice about cancer of the uterine cervix among women living in Kinshasa, the Democratic Republic of Congo

**DOI:** 10.1186/1472-6874-14-30

**Published:** 2014-02-18

**Authors:** Catherine Ali-Risasi, Paul Mulumba, Kristien Verdonck, Davy Vanden Broeck, Marleen Praet

**Affiliations:** 1 Laboratory of Anatomopathology, General Reference Hospital of Kinshasa, Kinshasa, Democratic Republic of Congo; 2 N.Goormaghtigh Institute of Pathology, Ghent University, De Pintelaan 185, B-9000 Ghent, Belgium; 3 Laboratory of Parasitology, University of Kinshasa (UNIKIN), Kinshasa, Democratic Republic of Congo; 4 Institute of Tropical Medicine Antwerp, Nationalestraat 155-200, B-2000 Antwerp, Belgium; 5 International Centre for Reproductive Health (ICRH), Ghent University, De Pintelaan 185, B-9000 Ghent, Belgium

## Abstract

**Background:**

Cervical cancer is the most frequent cancer of women in the Democratic Republic of Congo (DRC). Nevertheless, the level of women’s awareness about cervical cancer is unknown. Knowledge, attitude and practice (KAP) are important elements for designing and monitoring screening programs. The study purpose was to estimate KAP on cervical cancer and to identify associated factors.

**Methods:**

A cross-sectional study was conducted in Kinshasa, DRC, including 524 women aged 16–78 years (median age 28; interquartile range 22–35). The women were interviewed at home by trained field workers using a standardized questionnaire. The women’s score on knowledge, attitude and practice were dichotomized as sufficient or insufficient. We used binary and multiple logistic regression to assess associations between obtaining sufficient scores and a series of socio-demographic factors: age, residence, marital status, education, occupation, religion, and parity**.**

**Results:**

The women’s score on knowledge was not significantly correlated with their score on practice (Spearman’s rho = 0.08; P > 0.05). Obtaining a sufficient score on knowledge was positively associated with higher education (adjusted odds ratio (OR) 7.65; 95% confidence interval (95% CI) 3.31-17.66) and formal employment (adjusted OR 3.35; 95% CI 1.85-6.09); it was negatively associated with being single (adjusted OR 0.44; 95% CI 0.24-0.81) and living in the eastern, western and northern zone of Kinshasa compared to the city centre. The attitude score was associated with place of residence (adjusted OR for east Kinshasa: 0.49; 95% CI 0.27-0.86 and for south Kinshasa: 0.48; 95% CI 0.27-0.85) and with religion (adjusted OR 0.55; 95% CI 0.35-0.86 for women with a religion other than Catholicism or Protestantism compared to Catholics). Regarding practice, there were negative associations between a sufficient score on practice and being single (adjusted OR 0.24; 95% CI 0.13-0.41) and living in the eastern zone of the city (adjusted OR 0.39; 95% CI 0.22-0.70). Although 84% of women had heard about cervical cancer, only 9% had ever had a Papanicolaou (Pap) smear test.

**Conclusions:**

This study shows a low level of knowledge, attitude and practice on cervical cancer among women in Kinshasa. Increasing women’s awareness would be a first step in the long chain of conditions to attain a lower incidence and mortality.

## Background

Cervical cancer is after breast cancer the second most common cancer in women worldwide, with a global estimation of about 530,000 new cases and 275,000 deaths each year [1-3]. The highest incidence is estimated to occur in developing countries including those in Sub-Saharan Africa [3-7]. In contrast to high-income countries where mortality caused by cervical cancer is on the 8-9^th^ place, it is the most frequent fatal cancer in women in Sub-Saharan Africa reviewed in [2,8,9]. The incidence and mortality are expected to increase over the next 20 years [10]. The high mortality has been linked to inadequate screening for precancerous lesions. Unlike many other cancers, cervical cancer can be prevented either by focusing on primary prevention of human papilloma virus (HPV) infection and/or secondary prevention based on the early detection and treatment of precancerous lesions before they progress to invasive cancer.

Multiple factors contribute to inefficient screening of cervical cancer in low-income countries, such as the inadequacy or inexistence of a national screening system, poorly developed health services, the low access of the impoverished population to health care, the lack of technical and laboratory expertise, and, in general, the lack of public awareness [11,12]. All these factors contribute to inefficient testing, late diagnosis and late treatment [12,13]. In low-income countries, access to correct information is additionally prevented by illiteracy, some religious beliefs, beliefs in witchcraft, and social inequities. In Sub-Saharan Africa, only a small percentage of women regularly participate in cervical cancer screening [14]. Most women only seek treatment and care in an advanced stage of cervical cancer, too late to stop the lethal progression of the disease, although cervical cancer can be easily detected at an early precancerous stage. In contrast to developing countries, the mortality in high-income countries has been declining constantly over the past three decades. This decline has been related to the implementation of national screening programs, most frequently based on cytology [15,16].

The few papers [17,18] that exist on cervical cancer in the Democratic Republic of Congo (DRC) indicate that cervical cancer is the most frequent gynaecological cancer. A national program for early detection and treatment of high-grade lesions does not exist. Estimations of the prevalence and incidence have to rely therefore on extrapolation of data from other African countries [2]. Extrapolation of mortality rates of cervical cancer in Sub-Saharan Africa [2] to the context of Kinshasa with 9 million habitants means that each year about 800 women will die of cervical cancer in Congo’s capital. The level of awareness on cervical cancer of the female population of Kinshasa is unknown. Nevertheless, it is an important parameter for the development and monitoring of a screening program and for the follow-up of an eventual vaccination against HPV in the future. Awareness studies in Sub-Saharan Africa have been performed mostly in health care workers living in an urban environment and in the perspective of a future vaccination program [19-30].

This paper aims to assess knowledge, attitude and practice (KAP) concerning cervical cancer in women of the general population of Kinshasa, and to investigate socio-demographic factors that might influence it.

## Methods

### Participants

**Figure 1:**
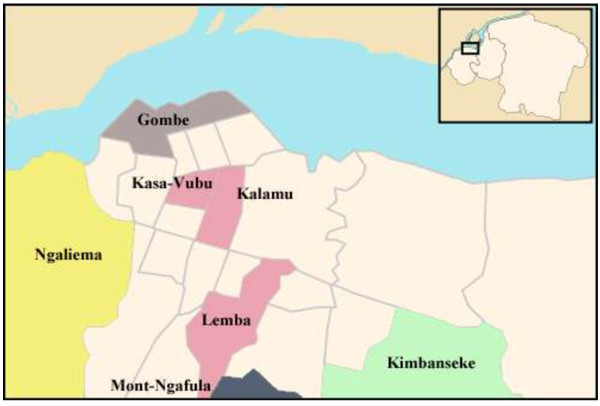
**Map of the city and the province of Kinshasa (source Wikipedia).** The figure illustrates the seven municipalities that were selected for the study. In the centre: Kalamu, Kasa-Vubu, and Lemba; in the east: Kimbanseke; in the west: Ngaliema; in the south: Mont-Ngafula; and in the north: Gombe.

The target population consisted of adult women living in Kinshasa. We grouped the 24 municipalities of Kinshasa in five zones: centre, east, west, south and north (Figure 1). We then took a sample of the target population in three steps: we randomly selected 7 out of the 24 municipalities (3 in the centre and 1 in each of the other 4 zones); in each selected municipality, we randomly selected one district; and in each selected district, we randomly selected three streets. Within the selected streets, the study interviewers systematically visited one out of ten lots (“*parcelles*”) and approached all the women who were present in these lots. The women were included if they were at least 16 years old, if they had been living in Kinshasa for six months, and if they were willing to participate in the study. Women who were not at home during the visit were not included.

The selected municipalities were Kimbanseke (eastern zone), Mont-Ngafula (southern zone), Ngaliema (western zone), Gombe (northern zone), and Kalamu, Kasa-Vubu and Lemba (city centre). Kimbanseke (eastern zone) is a relatively recent settlement in the hills. It is thought to be the most extended and the most populated municipality of the capital. Mont Ngafula (southern zone) is also a rather recent settlement in a hilly area. Mont Ngafula is a rapidly growing municipality. The municipality of Ngaliema accommodates several important compounds such as the residence of two former presidents, a military zone, and an exclusive quarter (*Mont Fleury*). Gombe (northern zone) is a smaller, residential and business area.

The number of participants from each of the five zones of Kinshasa was proportional to the demographic weight of that zone. Because of the descriptive nature of this study and because of the unavailability of previous information about the topic, we did not formally calculate a sample size. We aimed for a study size of 524 participants and expected that this would allow us to improve our understanding of the knowledge, attitude and practice of the women in Kinshasa. The recruitment period was from November to December 2008.

### Data collection and management

The outcome of interest was the level of knowledge, attitude and practice (dependent variables). The independent variables were: age, place of residence, marital status, education level, occupation, religion, and parity. KAP was assessed through a series of questions (Tables 1, 2, 3 and Additional files 1 and 2).

**Table 1:** Survey questions, and absolute and relative (%) number of expected answers on knowledge

**Questions**	**Expected answer**	**N (%) with score = 1**
1. Which diseases of the female genital tract do you know?	Description of at least one of the following diseases: leucorrhoea, infections, cancer of the uterine cervix, ovarian cysts or tumours, uterine myomas or tumours, dysmenorrhea, galactorrhoea	503 (96.0) ^1^
2. Have you ever heard about cervical cancer?	Yes	429 (81.9)
3. How did you hear about it?	Oral communication, newspaper, television, radio, conference, medical doctor or at the hospital, at a church or a school, or through a non-governmental organisation	434 (82.8)
4. What are the causes of cervical cancer?	Many sexual partners, use of plants for intimate care, sexually transmitted diseases, HIV infection, papilloma virus, old age	101 (19.3)
5. In your close circle of acquaintances, do you know someone who has had cervical cancer?	Yes	79 (15.1)
6. How can cervical cancer be treated?	Surgery, chemotherapy, radiotherapy	90 (17.2)
7. How can you prevent cervical cancer?	Avoid multiple sexual partners, avoid HIV infection, use condoms	92 (17.6)
8. Have you ever heard about cervical smears?	Yes	88 (16.8)
9. Do you know that suspect lesions can be detected early?	Yes	303 (57.8)

^1^241 participants (46.0%) spontaneously mentioned three or more items from the list and got 3 points; 126 (24.0%) gave two items and got 2 points; and 136 (26.0%) gave one item and got one point. The remaining 21 women (4.0%) did not describe any of the listed diseases.

**Table 2:** Survey questions, and absolute and relative (%) number of expected answers on attitude

**Questions**	**Expected answer**	**N (%) with score = 1**
1. What would you do in case of vaginal bleeding between periods?	Consult a medical doctor or go to a health centre	419 (80.0)
2. Are you willing to regularly consult a medical doctor for screening of cervical cancer?	Yes	297 (56.7)
3. Are you willing to get a smear test?	Yes	417 (79.6)
4. Would you want that a screening national program would be made available in the future?	Yes	498 (95.0)
5. Are you willing to pay for a smear test?	Yes	166 (31.7)

**Table 3:** Survey questions, and absolute and relative (%) number of expected answers on practices

**Questions**	**Expected answer**	**N (%) with score = 1**
1. When was your last gynaecological exam?	Less than 2 years ago	356 (67.9)
2. Do you use chemicals or plants for your intimate care?	No	372 (71.0)
3. Do you smoke?	No, never	507 (96.8)
4. How many sexual partners have you had in the last year?	Maximum one	456 (87.0)
5. Does your partner have a partner beside you?	No	195 (37.2)
6. Have you ever got a Pap smear test?	Yes	45 (8.6)

To standardize the application of the questionnaires, we organized three workshops in which candidate interviewers could familiarize with the questionnaire and its translation in Lingala, the most commonly used language in Kinshasa. Five interviewers were finally selected. Two independent data clerks entered all data in a Microsoft Excel file. A third person checked and corrected the discordances.

### Statistical analysis

Twenty items on the questionnaire were used to define the levels of knowledge, attitude and practice. For each item, a participant’s answer was considered to be correct if she gave at least one of the expected answers. This gave her one to three points. Detailed information on the scoring system is given in Table 1 and in Additional files 1 and 2. The maximum score for the questions on knowledge was 11, for attitude 5 and for practice 6.

The first analysis explored the correlations between the women’s scores on knowledge, attitude and practice. We calculated Spearman’s rank correlation coefficient (rho) and the corresponding P values for the correlation between the score on knowledge and the score on attitude, between knowledge and practice, and between attitude and practice.

The second analysis focused on the association between socio-demographic variables and the women’s scores. To facilitate this evaluation, the women’s scores (on an ordinal scale) were converted to binary variables (sufficient/insufficient score). The definition of a “sufficient” score was based on the participants’ median score. Women with a score of 6 or more on knowledge were categorized as having “sufficient” knowledge; the others had “insufficient” knowledge. For attitude and practice, the cut-off point was 4. In that way, approximately half of the women were grouped in the “sufficient” categories.

Binary logistic regression was used to explore the bivariate associations between the socio-demographic variables on the one hand and sufficient levels of knowledge, attitude and practice on the other hand. The women’s age was first described in five-year groups and then included in the logistic regression analyses in ten-year groups.

Multiple logistic regression models were developed separately for knowledge, attitude and practice. In a first step, all independent variables with a P-value of <0.2 on bivariate analysis were included in the logistic regression models. Next, we assessed if the remaining variables (with a P-value of ≥0.2 on bivariate analysis) could have a relevant effect on the models by including them one by one. Effect measure modification was evaluated by including the following interaction terms: place of residence × education, place of residence × occupation, and education × religion. Because the information on parity was not complete, we evaluated all multivariable analyses with and without this variable. Where more than one multiple logistic regression model was evaluated, we presented the model that best fitted the data.

The results of all logistic regression analyses are reported as odds ratios (OR) with 95% confidence intervals (95% CI). Excel and Stata/IC 10.1 were used for data analysis.

### Ethical considerations

The study protocol was approved by the Ethics Committee of the School of Health at Kinshasa University. All study participants gave oral informed consent.

## Results

### Participants

Five hundred twenty-four women participated in the study. Their median age was 28 years (interquartile range 22 to 35 years). The majority of women were single (54.2%), had a level of education of secondary school (58.2%), had no formal employment (57.5%), and adhered to religions other than Catholicism and Protestantism (58.8%), (Table 4). These other religions included several different groups of “*églises de réveil*”, the Kibanguist Church, and the Islam among other groups. About one third of the participants (37.3%) had given birth to three or more children.

**Table 4:** Socio-demographic characteristics of respondents

	**Place of residence in Kinshasa**					
	**Centre**	**East**	**West**	**South**	**North**	**Total**
	**(N = 224)**	**(N = 75)**	**(N = 75)**	**(N = 75)**	**(N = 75)**	**(N = 524)**
**Characteristic**	N (%)	N (%)	N (%)	N (%)	N (%)	N (%)
Age, *years*						
15-19	23 (10.3)	20 (26.7)	8 (10.7)	5 (6.7)	9 (12.0)	65 (12.4)
20-24	53 (23.7)	12 (16.0)	24 (32.0)	15 (20.0)	14 (18.7)	118 (22.5)
25-29	41 (18.3)	12 (16.0)	11 (14.7)	22 (29.3)	18 (24.0)	104 (19.8)
30-34	40 (17.9)	16 (21.3)	12 (16.0)	16 (21.3)	10 (13.3)	94 (17.9)
35-39	27 (12.1)	3 (4.0)	10 (13.3)	8 (10.7)	8 (10.7)	56 (10.7)
40-44	9 (4.0)	7 (9.3)	3 (4.0)	4 (5.3)	7 (9.3)	30 (5.7)
45-49	11 (4.9)	2 (2.7)	3 (4.0)	4 (5.3)	3 (4.0)	23 (4.4)
50-54	6 (2.7)	3 (4.0)	1 (1.3)	1 (1.3)	1 (1.3)	12 (2.3)
55-59	7 (3.1)	0 (0)	0 (0)	0 (0)	3 (4.0)	10 (1.9)
60-64	4 (1.8)	0 (0)	0 (0)	0 (0)	2 (2.7)	6 (1.1)
65-69	1 (0.4)	0 (0)	0 (0)	0 (0)	0 (0)	1 (0.2)
70-74	0 (0)	0 (0)	2 (2.7)	0 (0)	0 (0)	2 (0.4)
75-79	2 (0.9)	0 (0)	1 (1.3)	0 (0)	0 (0)	3 (0.6)
Marital status						
Married	86 (38.9)	34 (45.3)	27 (36.0)	29 (40.8)	26 (34.7)	202 (39.1)
Single	119 (53.8)	36 (48.0)	43 (57.3)	40 (56.3)	42 (56.0)	280 (54.2)
Widowed	8 (3.6)	2 (2.7)	3 (4.0)	1 (1.4)	3 (4.0)	17 (3.3)
Divorced	8 (3.6)	3 (4.0)	2 (2.7)	1 (1.4)	4 (5.3)	18 (3.5)
Level of education						
No school & primary	39 (17.7)	23 (30.7)	18 (24.0)	17 (23.0)	8 (10.7)	105 (20.2)
Secondary	128 (58.2)	50 (66.7)	47 (62.7)	46 (62.2)	31 (41.3)	302 (58.2)
Higher	53 (24.1)	2 (2.7)	10 (13.3)	11 (14.9)	36 (48.0)	112 (21.6)
Occupation						
No formal employment	124 (56.1)	57 (76.0)	48 (64.0)	49 (66.2)	21 (28.0)	299 (57.5)
Student	59 (26.7)	12 (16.0)	14 (18.7)	8 (10.8)	30 (40.0)	123 (23.7)
Formal employment	38 (17.2)	6 (8.0)	13 (17.3)	17 (23.0)	24 (32.0)	98 (18.8)
Religion						
Catholic	70 (31.4)	8 (10.7)	15 (20.0)	24 (32.4)	32 (42.7)	149 (28.5)
Protestant	22 (9.9)	7 (9.3)	10 (13.3)	13 (17.6)	14 (18.7)	66 (12.6)
Other	131 (58.7)	60 (80.0)	50 (66.7)	37 (50.0)	29 (38.7)	307 (58.8)
Parity^1^						
0 births	42 (24.9)	16 (21.9)	27 (36.0)	5 (10.9)	32 (45.1)	122 (28.1)
1 to 2 births	59 (34.9)	29 (39.7)	26 (34.7)	19 (41.3)	17 (23.9)	150 (34.6)
3 or more births	68 (40.2)	28 (38.4)	22 (29.3)	22 (47.8)	22 (31.0)	162 (37.3)

^1^The proportion of missing values was <2% for all variables except for parity (17% missing values).

### Scores

The participants’ median score on knowledge was 5 on a scale with a maximum of 11 (interquartile range 4–7); the median attitude score was 4 on a scale of 5 (interquartile range 3–4); and the median practice score was 4 on a scale of 6 (interquartile range 3–4). We found a weak but significant correlation between the women’s score on knowledge and attitude (r = 0.27, p < 0.001), and between attitudes and practice (r = 0.15, p < 0.001). No significant correlation was found between knowledge and practice (r = 0.08, p = 0.08). We used a cut-off of ≥6 to define a “sufficient” score on knowledge, ≥4 for attitude, and ≥4 for practice. In that way, 43% of the women obtained a sufficient score on knowledge, 52% on attitude, and 60% on practice.

### Knowledge

The women were first asked which diseases of the female genital organs they knew. The diseases that were reported most frequently were infections (mentioned by 83.6% of the women), myoma (mentioned by 47.9%), dysmenorrhea (43.1%) and cysts of the ovaries (37.2%). Cervical cancer came on the seventh place of this list; it was mentioned spontaneously by 64 women (12.2%). When the interviewer then specifically enquired about cervical cancer, the majority of the women (81.9%) told that they had heard about it, mostly through conversations with other people (73.4%) and less frequently through the media (30.3%). Only 3.7% got the information from a physician, a medical centre or a hospital. A minority of women (15.1%) knew a person in their neighbourhood who became victim to the disease. Fifteen percent of the women considered that having multiple sexual partners is an underlying cause of cervical cancer. Only 16.8% had ever heard of cervical smears. Table 1 lists the questions on knowledge, the expected answers, and the proportion of the participants who gave the expected answer.

On bivariate analysis, seven socio-demographic characteristics were significantly associated with having a sufficient level of knowledge: age, place of residence, marital status, education, occupation, religion and parity (Table 5). Women of 30–59 years old were more likely to get a sufficient score than the youngest group, with the best score in the age group of 50–59 years (OR 3.77; 95% CI: 1.38-10.34). Women from the eastern and the western parts of the city scored worse than the women from the city centre (OR east: 0.33; 95% CI: 0.18-0.60; OR west: 0.29; 95% CI: 0.16–0.53). Single women were less likely than married women to obtain a sufficient score (OR: 0.60; 95% CI: 0.42-0.87). There was a strong association with the level of education: highly educated women scored notably better than those who never went to school or only went to primary school (OR 4.41; 95% CI: 2.49-7.81). Also the women with a formal income scored well (OR: 3.18; 95% CI: 1.98-5.11) compared to the women without formal income. The women adhering to religions other than Catholicism and Protestantism were less likely to obtain a sufficient score compared to those who declared that they were Catholic (OR: 0.54; 95% CI: 0.37-0.81). Finally, women who had given birth to three or more children scored better than the nulliparous women (OR: 1.77; 95% CI: 1.09-2.87).

**Table 5:** Association between socio-demographic characteristics and knowledge

		**N (%) with score ≥6**^**1**^	**Crude OR**	**95% CI**	**Adjusted OR**^**2**^	**95% CI**
Age, *years*	10 – 19	18 (27.7)	1.00^**^		1.00	
	20 – 29	80 (36.0)	1.47	0.80 - 2.70	0.56	0.26 - 1.22
	30 – 39	80 (53.3)	2.98	1.59 - 5.61	1.52	0.62 - 3.71
	40 – 49	28 (52.8)	2.92	1.36 - 6.29	1.33	0.46 - 3.86
	50 – 59	13 (59.1)	3.77	1.38 - 10.34	1.56	0.39 - 6.27
	60 – 69	4 (57.1)	3.48	0.71 - 17.11	1.19	0.17 - 8.62
	70 – 79	2 (40.0)	1.74	0.27 - 11.29	2.53	0.22 - 28.54
Residence	Centre	109 (48.7)	1.00^***^		1.00	
	East	18 (24.0)	0.33	0.18 - 0.60	0.47^**^	0.24 - 0.90
	West	16 (21.3)	0.29	0.16 - 0.53	0.27^***^	0.13 - 0.55
	South	45 (60.0)	1.58	0.93 - 2.69	2.13^**^	1.14 - 3.99
	North	37 (49.3)	1.03	0.61 - 1.73	0.51^**^	0.27 - 0.96
Marital status	Married	101 (50.0)	1.00^**^		1.00	
	Single	105 (37.5)	0.60	0.42 - 0.87	0.44^**^	0.24 - 0.81
	Widowed	10 (58.8)	1.43	0.52 - 3.90	1.23	0.33 - 4.50
	Divorced	6 (33.3)	0.50	0.18 - 1.38	0.50	0.15 - 1.69
Education	No school/primary	38 (36.2)	1.00^***^		1.00	
	Secondary	104 (34.4)	0.93	0.58 - 1.47	1.15	0.65 - 2.01
	Higher	80 (71.4)	4.41	2.49 - 7.81	7.65^***^	3.31 - 17.66
Occupation	Non-formal income	105 (35.1)	1.00^***^		1.00	
	Student	57 (46.3)	1.60	1.04 - 2.44	1.60	0.79 - 3.26
	Formal income	62 (63.3)	3.18	1.98 - 5.11	3.35^***^	1.85 - 6.09
Religion	Catholic	76 (51.0)	1.00^**^		1.00	
	Protestant	37 (56.1)	1.23	0.68 - 2.19	1.40	0.70 - 2.81
	Other	111 (36.2)	0.54	0.37 - 0.81	0.93	0.57 - 1.51
Parity	0 births	42 (34.4)	1.00^**^			
	1 to 2 births	54 (36.0)	1.07	0.65 - 1.77		
	3 or more births	78 (48.1)	1.77	1.09 - 2.87		

OR: odds ratio; crude OR: odds ratio by bivariate analysis.

95% CI: confidence interval at the 95% level.

^*^: 0.05 ≤ p < 0.2; ^**^: 0.001 ≤ p < 0,05; ^***^: p < 0.001.

^1^Absolute number and proportion of women belonging to a specific category who got a sufficient score on knowledge, e.g. out of 65 women aged 10 to 19 years, 18 (27.7%) had a score of ≥6 points.

^2^Adjusted OR: odds ratio by multiple logistic regression (variables in the model: age, residence, marital status, education, occupation, and religion).

On multivariable analysis, the positive associations of higher education (adjusted OR 7.65; 95% CI 3.31-17.66) and a regularly paid job (adjusted OR 3.35; 95% CI 1.85-6.09) with sufficient knowledge remained significant. Also the negative associations of being single (adjusted OR 0.44; 95% CI 0.24-0.81) or living in the eastern or western part of Kinshasa with sufficient knowledge remained significant (OR east: 0.47; 95% CI: 0.24-0.90; OR west: 0.27; 95% CI: 0.13-0.55, compared to the centre of the city). In addition, the women from the north were less likely to obtain a sufficient score (OR: 0.51; 95% CI: 0.27-0.96).

The product terms residence × education, residence × occupation, and education × religion were not significant and were not included in the final model. The inclusion of the variable parity in the multivariable logistic regression model did not change the findings: there was no significant association between a sufficient knowledge score and parity (OR: 0.95; 95% CI: 0.46-1.89) for three or more compared to zero births), and the other associations (with place of residence, marital status, education, and occupation) remained significant.

### Attitude

Table 2 lists the five questions we used to assess the women’s attitude. Most of the respondents (80.0%) were willing to consult a medical doctor in case of abnormal bleeding between menstruations. In addition, 56.7% told the interviewers that they were willing to regularly consult a physician for screening of cervical cancer. Eighty percent were willing to have a smear test and 95% were in favour of the installation of a national screening program. Although the majority wanted to be screened, only 31.7% was prepared to pay for it.

On bivariate analysis, four factors were significantly associated with a sufficient score on attitude: age, place of residence, education and religion. As for knowledge, the age group of 50–59 years scored best. Women from the eastern, western and southern part of the city scored worse than those from the centre (OR east: 0.36; 95% CI: 0.21-0.63; OR west: 0.54; 95% CI: 0.32-0.91; OR south: 0.48; 95% CI: 0.28-0.82). Among those with a sufficient attitude score, there were more women with higher education (OR 1.83; 95% CI 1.07-3.16) and fewer women of a religion other than Catholicism or Protestantism (OR 0.49; 95% CI 0.33-0.73),

On multivariable analysis, the place of residence and religion remained significant. Women from the eastern and southern zone of the city were less likely to have a sufficient score on attitude than those from the centre (adjusted OR east: 0.49; 95% CI 0.27-0.86; adjusted OR south 0.48; 95% CI: 0.27-0.85) (Table 6). Those women who declared to have a religion other than Catholicism or Protestantism were also less likely to get a sufficient attitude score (adjusted OR 0.55; 95% CI 0.35–0.86 compared to adherents of the Catholic Church).

**Table 6:** Association between socio-demographic characteristics and attitude

		**N (%) with score ≥4**^**1**^	**Crude OR**	**95% CI**	**Adjusted OR**^**2**^	**95% CI**
Age, *years*	10 - 19	23 (35.4)	1.00^*^		1.00	
	20 - 29	116 (52.3)	2.00	1.13 - 3.54	1.50	0.78 - 2.87
	30 - 39	82 (54.7)	2.20	1.21 - 4.02	1.74	0.84 - 3.57
	40 - 49	30 (56.6)	2.38	1.31 - 5.01	2.00	0.85 - 4.72
	50 - 59	15 (68.2)	3.91	1.40 - 10.97	2.57	0.80 - 8.25
	60 - 69	4 (57.1)	2.43	0.50 - 11.83	0.87	0.15 - 5.13
	70 -79	2 (40.0)	1.22	0.19 - 7.82	0.34	0.03 - 3.88
Residence	Centre	136 (60.7)	1.00^**^		1.00	
	East	27 (36.0)	0.36	0.21 - 0.63	0.49^**^	0.27 - 0.86
	West	34 (45.3)	0.54	0.32 - 0.91	0.64	0.37 - 1.12
	South	32 (42.7)	0.48	0.28 - 0.82	0.48^**^	0.27 - 0.85
	North	43 (57.3)	0.87	0.51 - 1.48	0.72	0.41 - 1.27
Marital status	Married	112 (55.4)	1.00			
	Single	135 (48.2)	0.75	0.52 - 1.08		
	Widowed	9 (52.9)	0.9	0.34 - 2.44		
	Divorced	11 (61.1)	1.26	0.47 - 3.39		
Education	No school/primary	52 (49.5)	1.00^**^		1.00	
	Secondary	143 (47.4)	0.92	0.59 - 1.43	1.01	0.61 - 1.65
	Higher	72 (64.3)	1.83	1.07 - 3.16	1.57	0.78 - 3.14
Occupation	Non-formal income	146 (48.8)	1.00		1.00	
	Student	64 (52.0)	1.14	0.75 - 1.73	0.93	0.52 - 1.68
	Formal income	59 (60.2)	1.59	1.00 - 2.52	1.30	0.78 - 2.17
Religion	Catholic	95 (63.8)	1.00^**^		1.00	
	Protestant	35 (53.0)	0.64	0.36 - 1.15	0.68	0.37 - 1.27
	Other	142 (46.3)	0.49	0.33 - 0.73	0.55^**^	0.35 - 0.86
Parity	0 births	58 (47.5)	1.00^*^			
	1 to 2 births	71 (47.3)	0.99	0.61 - 1.60		
	3 or more births	94 (58.0)	1.53	0.95 - 2.45		

OR: odds ratio; crude OR: odds ratio by bivariate analysis.

95% CI: confidence interval at the 95% level.

*: 0.05 ≤ p < 0.2; **: 0.001 ≤ p < 0,05; ***: p < 0.001.

^1^Absolute number and proportion of women belonging to a specific category who got a sufficient score on attitude, e.g. out of 65 women aged 10 to 19 years, 23 (35.4%) had a score of ≥4 points.

^2^Adjusted OR: odds ratio by multiple logistic regression (variables in the model: age, residence, education, occupation, and religion).

The inclusion of product terms (residence × education, residence × occupation, and education × religion) and of additional variables (parity and marital status) in the multiple logistic regression model did not change the findings. On multivariable analysis, there was no significant association between parity and having a sufficient attitude score (OR: 1.66; 95% CI: 0.84-3.28 for three or more compared to zero births).

### Practice

Concerning practice (Table 3), 67.9% of the participants had undergone a gynaecological examination during the last two years. The majority (71.0%) declared not to use plants or chemicals for their intimate care, and not to smoke (96.8%). Most women (87.0%) declared to have had sexual contacts with maximum one partner during the last year, and 37.2% were convinced that their partner had no other sexual contacts. Only 8.6% had already had a Pap test.

On bivariate analysis, four socio-demographic factors were significantly associated with a sufficient score on practice: place of residence, marital status, occupation and parity. The women who lived in the eastern and northern part of the city scored worse than those from the centre (OR east: 0.48; 95% CI: 0.28-0.82; OR north: 0.57; 95% CI: 0.33-0.96). In addition, single women were less likely to obtain a sufficient score than married women (OR: 0.31; 95% CI: 0.21-0.46). Finally, the women who had given birth to three or more children scored better than the nulliparous women (OR: 2.59; 95% CI: 1.57-4.28).

The associations with place of residence and marital status remained significant on multivariable analysis (Table 7). The women living in the east of the city were less likely than those from the centre to obtain a sufficient score on practice (adjusted OR 0.39; 95% CI 0.22-0.70). There was also a negative association between being single (adjusted OR: 0.24; 95% CI: 0.13-0.41) or being widow (adjusted OR 0.22; 95% CI: 0.06–0.86) and obtaining a sufficient practice score.

**Table 7:** Association between socio-demographic characteristics and practice

		**N (%) with score ≥4**^**§**^	**Crude OR**	**95% CI**	**Adjusted OR**	**95% CI**
Age, *years*	10 – 19	35 (53.8)	1.00^*^		1.00	
	20 – 29	124 (55.9)	1.08	0.62 - 1.89	0.74	0.40 - 1.35
	30 – 39	93 (62.0)	1.40	0.78 - 2.52	0.41^**^	0.19 - 0.89
	40 – 49	35 (66.0)	1.67	0.79 - 3.52	0.47	0.18 - 1.22
	50 – 59	18 (81.8)	3.86	1.18 - 12.65	1.66	0.36 - 7.75
	60 – 69	6 (85.7)	5.14	0.59 - 45.15	1.05	0.10 - 10.59
	70 -79	4 (80.0)	3.43	0.36 - 32.36	1.54	0.11 - 21.00
Residence	Centre	147 (60.7)	1.00^**^		1.00	
	East	36 (48.0)	0.48	0.28 - 0.82	0.39^**^	0.22 - 0.70
	West	47 (62.7)	0.88	0.51 - 1.51	0.92	0.52 - 1.63
	South	46 (61.3)	0.83	0.48 - 1.43	0.98	0.54 - 1.79
	North	39 (52.0)	0.57	0.33 - 0.96	0.58	0.33 - 1.04
Marital status	Married	153 (75.7)	1.00^***^		1.00	
	Single	138 (49.3)	0.31	0.21 - 0.46	0.24^***^	0.13 - 0.41
	Widowed	10 (58.8)	0.46	0.17 - 1.27	0.22^**^	0.06 - 0.86
	Divorced	11 (61.1)	0.50	0.19 - 1.37	0.50	0.18 - 1.42
Education	No school/primary	63 (60.0)	1.00			
	Secondary	185 (61.3)	1.05	0.67 - 1.66		
	Higher	63 (56.3)	0.86	0.50 - 1.47		
Occupation	Non-formal income	194 (64.9)	1.00^**^		1.00	
	Student	59 (48.0)	0.50	0.33 - 0.76	0.77	0.45 - 1.31
	Formal income	59 (60.2)	0.82	0.51 - 1.31	1.09	0.64 - 1.85
Religion	Catholic	88 (59.1)	1.00			
	Protestant	43 (65.2)	1.30	0.71 - 2.37		
	Other	182 (59.3)	1.01	0.68 - 1.50		
Parity	0 births	56 (53.3)	1.00^***^			
	1 to 2 births	84 (56.0)	1.12	0.69 - 1.80		
	3 or more births	121 (74.7)	2.59	1.57 - 4.28		

OR: odds ratio; crude OR: odds ratio by bivariate analysis.

95% CI: confidence interval at the 95% level.

*: 0.05 ≤ p < 0.2; **: 0.001 ≤ p < 0,05; ***: p < 0.001.

^1^Absolute number and proportion of women belonging to a specific category who got a sufficient score on practice, e.g. out of 65 women aged 10 to 19 years, 35 (53.8%) had a score of ≥4 points.

^2^Adjusted OR: odds ratio by multiple logistic regression (variables in the model: age, residence, marital status, and occupation).

The inclusion of product terms and of additional variables (education, religion, and parity) in the multiple logistic regression model did not change the findings. On multivariable analysis, there was no significant association between parity and having a sufficient practice score (OR: 1.61; 95% CI: 0.75-3.48 for three or more compared to zero births).

## Discussion

The present study explored the knowledge, attitude, and associated behaviours of women in Kinshasa with regard to cancer of the uterine cervix. The results provide support for the view that among African women, the awareness about cervical cancer, and especially its prevention and treatment, is very limited. Of note, several socio-demographic parameters (place of residence, level of education, occupation, marital status, and religion) were found to affect the women’s knowledge, attitude, and/or practice (KAP).

Several limitations of the present study should be acknowledged. Firstly, although we believe that the houses that were visited are fairly representative of the houses in Kinshasa, selection bias may still affect the internal validity of the study. The main reason for this is that our study population did not include women who were not at home when the interviewers visited their houses nor did it include women who did not want to participate. Secondly, as noted by Launiala [31], there are some limitations to the KAP survey method itself. These limitations include the difficulty of expressing concepts of western medicine in a local African language, the possibility of social desirability bias (i.e. the tendency of interviewees to over-report what they believe to be “good behaviour”), and the fact that the KAP survey is not a good method to obtain in-depth information about sensitive issues. In spite of the limitations, this study stands as a first attempt to systematically explore and document KAP about cervical cancer among women in the DRC.

Our study shows that, although only 12% of the women of Kinshasa spontaneously mentioned cervical cancer as one of the diseases of the female genital organs, the large majority (82%) of these women reported to have heard about the disease. These results are similar to those published for Ethiopia (79%) [32], Ghana (93%) [33], and Nigeria (71%) [34]. Lower figures have been published for Botswana (23%) [35] and in other reports for Nigeria (about 48%) [36-38]. In most of these studies, the concept of “knowledge” was very elementary and limited to a positive answer on ‘having heard’ about cancer. With regard to the knowledge of risk factors, 15% of the respondents related cervical cancer to sexual behaviour. This finding agrees with observations in Nigeria [37], Ethiopia [32] and Botswana [39].

Although newspapers, radio and television are easily accessible in Kinshasa, word-of-mouth communication was the most important source of information. This finding is consistent with a publication from Botswana [35], but contrasts with studies in Ethiopia [32] and Nigeria [37] where the media came on the first place. But in these countries too, oral communication remained an important way of exchange of information on health issues. In rural areas without electricity, it is the only way of communication.

The population of Kinshasa (about 9 million) is estimated to represent 12% of the total population (about 75 million) of the DRC. The majority of the Congolese population live in rural areas where electricity is not always available and information transfer via the media is scarce. The results of our study are limited to the urban population of the capital which is favoured by easy access to information, and even within Kinshasa, we found that the place of residence was associated with the women’s knowledge about cervical cancer. The relative role of the media in the transfer of knowledge may become more important when governments will use them more actively in a policy of cancer prevention. Furthermore, because of the oral culture, churches may play an important role in the transfer of information on health care matters.

About half of the women (58%) told the interviewers that they knew that suspect lesions can be detected early. However, the proportion of women who had heard about cervical smear testing was substantially lower (17%), and only 9% of the participants had ever had a Pap test. This finding is consistent with the low effective coverage rates for cervical cancer screening in developing countries reported elsewhere: on average 19% compared to 63% in high-income countries [14]. The situation might even be worse, as a recent survey in 15 developing countries has indicated, that as few as 4% of the women aged 18 – 69 years had undergone a pelvic exam or a Pap smear test in the past three years [40]. Poor access to health care in general, and the lack of specific screening and treatment facilities in particular may explain the low coverage rates for cervical cancer screening in developing countries [40].

Concerning attitude, a large majority of women (80%) said that they agreed to participate in a screening program, but considerably less women (30%) would be willing to pay for a smear test. This suggests that financial issues should be taken into consideration when a wide-scale screening program is introduced in Kinshasa. Nowadays, the cost of a Pap smear in Kinshasa varies between 30 US$ and 50 US$. Most women in an impoverished society who gain 1 US$ to 2 US$ a day are unable to pay this amount. Part of this high cost is related to the lack of trained people to read and interpret the Pap smears, the insufficient number of tests performed, and the low appreciation of preventive measures as a profitable strategy at the long end. If screening is already an obstacle because of the high cost, it is difficult to motivate women to pay even more for preventive treatment such as loop electrical excision procedure or cryotherapy.

The correlations between the knowledge and attitude scores and between attitude and practice were significant but weak, and the correlation between knowledge and practice was even not significant. This finding adds to the growing body of evidence showing that increased knowledge is not automatically translated into changes in attitude and practice. Some studies have even found that a high level of awareness, as encountered in medical students and physicians, was associated with a lower level of practice [34,37,41,42]. Even in countries where information about cervical cancer is easily available, the effective coverage of screening was found to be only 63% [14].

By multivariable analysis, we found that knowledge was associated with four socio-demographic factors: the place of residence within Kinshasa, level of education, occupation and marital status. The municipality of Mont-Ngafula in south Kinshasa had a high proportion of women with a sufficient score on knowledge. Mont-Ngafula is semi-residential area that is situated near the University of Kinshasa and the Higher Institute for Medical Technology and Nursing. It may be expected that women living in these places have better access to information than the women living elsewhere. They are also likely to have received a better education and to have better jobs. However, the effect of the place of residence on knowledge cannot be explained by education and employment alone, because these factors were included in the multivariable analysis. Therefore, other factors that are still unknown are likely to explain the association between the place of residence and the level of knowledge.

In our study, married women scored better on knowledge than singles. Contradictory reports have been published on the influence of marital status on knowledge: in a Nigerian study, singles scored better for the awareness on cervical cancer [37] while in an Ethiopian study, no effect was found [32]. Overall, the participants who were most likely to have a sufficient knowledge score were the married women with a degree of higher education, a regular income and living in the southern district of Mont-Ngafula.

With regard to attitude, the significantly associated factors on multivariable analysis were the place of residence and religion. Although the women from Mont Ngafula scored well on knowledge, this was not reflected in their attitude scores. They were less likely to get a sufficient score than the women from the city centre, illustrating the poor correlation between knowledge and attitude. In this study, adherents to the Catholic Church scored better than the category ‘other religions’. The category ‘other’ included a multitude of small and diverse churches which were difficult to classify in one well-defined group. Most of the women in this group adhered to the “*églises de réveil*” and the Kibanguist church. Religion, however, was not associated with a better practice.

Regarding practice, marital status and place of residence were independent predictors of a sufficient score. Married women were more likely than single women to obtain a sufficient score, and women from eastern Kinshasa (Kimbanseke) scored worse on practice compared to the women from the city centre. Kimbanseke is an extended municipality with a large, and relatively recent population. A high proportion (25/75) of the women from this municipality had insufficient scores on knowledge, attitude as well as practice.

Information on the level of KAP and the socio-demographic factors influencing it is important to guide future awareness campaigns about cervical cancer. The fact that we found no significant associations between parity and KAP suggests that women did not learn about cervical cancer when they had contact with health services. Most women in the RDC give birth in health care centres and are assisted by skilled health personnel [43]. This could be considered therefore to be a missed opportunity in order to increase the awareness about cervical cancer through the existing health services. The fact that only 4% of the women mentioned doctors or health services as a source of information about cervical cancer is an additional illustration of this missed opportunity. In addition to health services, local churches appear to be a relevant stakeholder in the organization of awareness campaigns, given our findings on the probable influence of religious groups on attitude and on the importance of oral communication as a source of information. The potential of church communities in the promotion of healthy behaviour has been widely recognized [44].

Finally, our study illustrates that women are clearly willing to participate in screening initiatives. Nevertheless, it is important to recognize that a screening strategy can only be useful if it improves patient-important outcomes. This is possible only if effective treatment is available, if test-related adverse events or anxiety are reduced, and/or if prognostic information improves patients’ wellbeing [11,45].

## Conclusions

Our findings underline the low level of knowledge, attitude and practice regarding cervical cancer among women in Kinshasa despite the high incidence of this cancer. Increasing the women’s awareness is an important first step in the long chain of conditions to attain a lower incidence and mortality. In parallel with an increased awareness, the national health care system should facilitate and encourage early diagnosis and therapy.

## Competing interests

The authors declare that they have no competing interests.

## Pre-publication history

The pre-publication history for this paper can be accessed here:

http://www.biomedcentral.com/1472-6874/14/30/prepub

## Supplementary Material

Additional file 1 Questionnaire. Click here for file

Additional file 2 Questionnaire, expected answers, and scoring system. Click here for file
