# Supplementary material for: Knowledge, attitude and practice about cancer of the uterine cervix among women living in Kinshasa, the Democratic Republic of Congo
Source: BMC Womens Health. 2014 Feb 18;14:30. doi: 10.1186/1472-6874-14-30 (PMC3937079; doi:10.1186/1472-6874-14-30)
Supplement: Additional file 1 — Questionnaire. [file 1472-6874-14-30-S1.docx]

Additional file 1:

Questionnaire

KNOWLEDGE

1. Which diseases of the female genital tract do you know?
2. Have you ever heard about cancer of the uterine cervix?
3. How did you hear about it?
4. What are the causes of cervical cancer?
5. In your close circle of acquaintances, do you know someone who has had cervical cancer?
6. How can cervical cancer be treated?
7. How can cervical cancer be prevented?
8. Have you ever heard about cervical smears?
9. Do you know that suspect lesions can be detected early?

ATTITUDE

1. What would you do in case of vaginal bleeding between periods?
2. Are you willing to regularly consult a medical doctor for screening of cervical cancer?
3. Are you willing to get a smear test?
4. Would you want that a screening national program would be made available in the future?
5. Are you willing to pay for a Pap smear test?

PRACTICE

1. When was your last gynaecological examination?
2. Do you use chemicals of plants for your intimate care?
3. Do you smoke?
4. How many sexual partners have you had in the last year?
5. Does your partner have a partner beside you?
6. Have you ever got a Pap smear test?
