# Supplementary material for: Knowledge, attitude and practice about cancer of the uterine cervix among women living in Kinshasa, the Democratic Republic of Congo
Source: BMC Womens Health. 2014 Feb 18;14:30. doi: 10.1186/1472-6874-14-30 (PMC3937079; doi:10.1186/1472-6874-14-30)
Supplement: Additional file 2 — Questionnaire, expected answers, and scoring system. [file 1472-6874-14-30-S2.docx]

Additional file 2:

Questionnaire, expected answers, and scoring system

KNOWLEDGE

1. Which diseases of the female genital tract do you know?

- Expected answers:
- leucorrhoea
- infections or sexually transmitted diseases
- cancer of the uterine cervix
- ovarian cysts
- uterine tumour or myoma
- dysmenorrhea
- galactorrhoea
- Scoring: 1 point for a description of 1 disease from the list, 2 points for a description of 2 diseases and 3 for descriptions of 3 diseases from the list

1. Have you ever heard about cancer of the uterine cervix?

- Expected answer: yes
- Scoring: 1 point if yes

1. How did you hear about it?

- Expected answers:
- oral communication
- newspaper
- television
- radio
- conference
- medical doctor or at the hospital
- church
- school
- non-governmental organisation
- Scoring: 1 point if at least one of the listed answers is given

1. What are the causes of cervical cancer?

- Expected answers:
- many sexual partners
- use of plants for intimate care
- sexually transmitted diseases
- HIV infection
- papilloma virus
- old age
- Scoring: 1 point if at least one of the listed answers is given

1. In your close circle of acquaintances, do you know someone who has had cervical cancer?

- Expected answer: yes
- Scoring: 1 point if yes

1. How can cervical cancer be treated?

- Expected answers:
- surgery
- chemotherapy
- radiotherapy
- Scoring: 1 point if at least one of the listed answers is given

1. How can cervical cancer be prevented?

- Expected answers:
- avoid multiple sexual partners
- avoid HIV infection
- use condoms
- vaccination
- Scoring: 1 point if at least one of the listed answers is given

1. Have you ever heard about cervical smears?

- Expected answer: yes
- Scoring: 1 point if yes

1. Do you know that suspect lesions can be detected early?

- Expected answer: yes
- Scoring: 1 point if yes

ATTITUDE

1. What would you do in case of vaginal bleeding between periods?

- Expected answers:
- consult a medical doctor
- go to a health centre
- Scoring: 1 point if at least one of the listed answers is given

1. Are you willing to regularly consult a medical doctor for screening of cervical cancer?

- Expected answer: yes
- Scoring: 1 point if yes

1. Are you willing to get a smear test?

- Expected answer: yes
- Scoring: 1 point if yes

1. Would you want that a screening national program would be made available in the future?

- Expected answer: yes
- Scoring: 1 point if yes

1. Are you willing to pay for a Pap smear test?

- Expected answer: yes, or a concrete amount
- Scoring: 1 point if yes or any amount >0 US$

PRACTICE

1. When was your last gynaecological examination?

- Expected answer: any moment in the past 2 years
- Scoring: 1 point if less than 2 years ago

1. Do you use chemicals of plants for your intimate care?

- Expected answer: no
- Scoring: 1 point if no products are used

1. Do you smoke?

- Expected answer: no
- Scoring: 1 point if the participant has never smoked (0 points for current or past smokers)

1. How many sexual partners have you had in the last year?

- Expected answer: maximum one
- Scoring: 1 point if maximum one partner in the last year

1. Does your partner have a partner beside you?

- Expected answer: no
- Scoring: 1 point if no

1. Have you ever got a Pap smear test?

- Expected answer: yes
- Scoring: 1 point if a smear test had been performed in the past
